# Supplementary material for: Functional Limitations and Use of General Health Examination and Cancer Screening Among People with Disabilities Who Need Support from Others: Secondary Data Analysis of the 2022 Comprehensive Survey of Living Conditions in Japan
Source: Int J Environ Res Public Health. 2025 Mar 24;22(4):484. doi: 10.3390/ijerph22040484 (PMC12026762; doi:10.3390/ijerph22040484)
Supplement: Supplementary file 1 [file ijerph-22-00484-s001.zip › Suppl1.pdf]

Table S1. The numbers and percentages of data with missing value among data which met the inclusion criteria of age (20 ≦ years old ≦ 74) and living place (living in house). (n=29,175; men=14,157, women=15,018). These data were removed from final analysis.

	Number of data with missing value	(%)
Outcome variables		
General health examination	161	(0.55)
Lung cancer screening	535	(1.83)
Colorectal cancer screening	474	(1.63)
Gastric cancer screening	525	(1.80)
Cervical cancer screening †	296	(1.97)
Breast cancer screening †	275	(1.83)
Explanatory variables		
Disability	0	(0)
Washington Group Short Set		
Vision	386	(1.32)
Hearing	1205	(4.13)
Mobility	434	(1.49)
Cognition	471	(1.61)
Self-care	433	(1.48)
Communication	412	(1.41)
Cofounding variables		
Sex	0	(0)
Age	0	(0)
Marital status	0	(0)
Educational qualification	3557	(12.19)
Constant visit to hospitals	134	(0.46)
Subjective health status	203	(0.70)
Alcohol consumption	351	(1.20)
Smoking habit	293	(1.00)
Subjective financial state	0	(0)
Kessler Psychological Distress Scale	759	(2.60)
Health insurance	246	(0.84)
Employment status	396	(1.36)

† Percentages were calculated for only women (n=15,018)
