# Supplementary material for: Functional Limitations and Use of General Health Examination and Cancer Screening Among People with Disabilities Who Need Support from Others: Secondary Data Analysis of the 2022 Comprehensive Survey of Living Conditions in Japan
Source: Int J Environ Res Public Health. 2025 Mar 24;22(4):484. doi: 10.3390/ijerph22040484 (PMC12026762; doi:10.3390/ijerph22040484)
Supplement: Supplementary file 1 [file ijerph-22-00484-s001.zip › Suppl2.pdf]

Table S2a. Participant characteristics stratified by participation or nonparticipation in the general health examination and lung cancer screening.

	General health examination (n=23,868)														Lung cancer screening (n=18,091)													
	Participants(n=17,911)							Non participant(n=5,957)							Participants (n=9,716)							Non participant (n=8375)						
	Number	Percent	(	95% CI			)	Number	Percent	(	95% CI			)	Number	Percent	(	95% CI			)	Number	Percent	(	95% CI			)
Disability																												
No-need any support or supervision	17686	98.74	(	98.57	−	98.90	)	5739	96.34	(	95.84	–	96.79	)	9600	98.81	(	98.58	–	99.01	)	8152	97.34	(	96.98	–	97.67	)
Need any support or supervision	225	1.26	(	1.10	–	1.43	)	218	3.66	(	3.21	–	4.16	)	116	1.19	(	0.99	–	1.42	)	223	2.66	(	2.33	–	3.02	)
WG sub questions (vision)																												
“No difficulty” or “some difficulty”	17367	96.96	(	96.70	–	97.21	)	5748	96.49	(	96.00	–	96.94	)	9409	96.84	(	96.48	–	97.17	)	8084	96.53	(	96.12	–	96.90	)
“A lot of difficulty” or “cannot do at all”	544	3.04	(	2.79	–	3.30	)	209	3.51	(	3.06	–	4.00	)	307	3.16	(	2.83	–	3.52	)	291	3.47	(	3.10	–	3.88	)
WG sub questions (hearing)																												
“No difficulty” or “some difficulty”	17564	98.06	(	97.85	–	98.26	)	5825	97.78	(	97.39	–	98.14	)	9529	98.08	(	97.79	–	98.33	)	8202	97.93	(	97.61	–	98.22	)
“A lot of difficulty” or “cannot do at all”	347	1.94	(	1.74	–	2.15	)	132	2.22	(	1.86	–	2.61	)	187	1.92	(	1.67	–	2.21	)	173	2.07	(	1.78	–	2.39	)
WG sub questions (mobility)																												
“No difficulty” or “some difficulty”	17406	97.18	(	96.93	–	97.42	)	5644	94.75	(	94.16	–	95.29	)	9441	97.17	(	96.83	–	97.49	)	7984	95.33	(	94.86	–	95.77	)
“A lot of difficulty” or “cannot do at all”	505	2.82	(	2.58	–	3.07	)	313	5.25	(	4.71	–	5.84	)	275	2.83	(	2.51	–	3.17	)	391	4.67	(	4.23	–	5.14	)
WG sub questions (remembering or concentrating)																												
“No difficulty” or “some difficulty”	17557	98.02	(	97.81	–	98.22	)	5750	96.53	(	96.04	–	96.97	)	9549	98.28	(	98.01	–	98.53	)	8166	97.50	(	97.15	–	97.82	)
“A lot of difficulty” or “cannot do at all”	354	1.98	(	1.78	–	2.19	)	207	3.47	(	3.03	–	3.96	)	167	1.72	(	1.47	–	1.99	)	209	2.50	(	2.18	–	2.85	)
WG sub questions (self-care)																												

“No difficulty” or “some difficulty”	17620	98.38	(98.18 – 98.55)	5798	97.33	(96.90 – 97.72)	9586	98.66	(98.42 – 98.88)	8200	97.91	(97.59 – 98.20)
“A lot of difficulty” or “cannot do at all”	291	1.62	(1.45 – 1.82)	159	2.67	(2.28 – 3.10)	130	1.34	(1.12 – 1.58)	175	2.09	(1.80 – 2.41)
WG sub questions (communication)												
“No difficulty” or “some difficulty”	17573	98.11	(97.91 – 98.30)	5765	96.78	(96.31 – 97.20)	9581	98.61	(98.36 – 98.83)	8194	97.84	(97.51 – 98.13)
“A lot of difficulty” or “cannot do at all”	338	1.89	(1.70 – 2.09)	192	3.22	(2.80 – 3.69)	135	1.39	(1.17 – 1.64)	181	2.16	(1.87 – 2.49)
Numbers of difficulty defined by WG												
0	16756	93.55	(93.18 – 93.90)	5367	90.10	(89.32 – 90.83)	9065	93.30	(92.79 – 93.78)	7633	91.14	(90.52 – 91.73)
1	754	4.21	(3.92 – 4.51)	352	5.91	(5.33 – 6.53)	452	4.65	(4.25 – 5.08)	481	5.74	(5.26 – 6.26)
2 and more	401	2.24	(2.03 – 2.46)	238	4.00	(3.52 – 4.52)	199	2.05	(1.78 – 2.34)	261	3.12	(2.76 – 3.50)
Sex												
Male	9118	50.91	(50.18 – 51.64)	2458	41.26	(40.02 – 42.52)	5032	51.79	(50.80 – 52.78)	3711	44.31	(43.25 – 45.38)
Female	8793	49.09	(48.36 – 49.82)	3499	58.74	(57.48 – 59.98)	4684	48.21	(47.22 – 49.20)	4664	55.69	(54.62 – 56.75)
Age (years)												
65–74	4171	23.29	(22.67 – 23.91)	1883	31.61	(30.44 – 32.80)	3027	31.15	(30.24 – 32.08)	2944	35.15	(34.13 – 36.18)
40–64	9629	53.76	(53.03 – 54.49)	2586	43.41	(42.16 – 44.67)	6689	68.85	(67.92 – 69.76)	5431	64.85	(63.82 – 65.87)
20–39	4111	22.95	(22.34 – 23.57)	1488	24.98	(23.89 – 26.09)	-	-	(- – -)	-	-	(- – -)
Marital status												
Married	12347	68.94	(68.25 – 69.61)	3634	61.00	(59.76 – 62.24)	7602	78.24	(77.41 – 79.05)	5928	70.78	(69.80 – 71.75)
Single	4000	22.33	(21.73 – 22.95)	1692	28.40	(27.27 – 29.56)	1129	11.62	(10.99 – 12.27)	1392	16.62	(15.84 – 17.43)
Divorced/widowed	1564	8.73	(8.33 – 9.15)	631	10.59	(9.83 – 11.39)	985	10.14	(9.55 – 10.75)	1055	12.60	(11.90 – 13.32)
Educational qualification												
Vocational school/junior college/community(technical) college/university/post graduate school	9714	54.23	(53.50 – 54.96)	2670	44.82	(43.56 – 46.09)	4956	51.01	(50.01 – 52.00)	3668	43.80	(42.74 – 44.86)
High school	7356	41.07	(40.35 – 41.79)	2697	45.27	(44.01 – 46.54)	4232	43.56	(42.57 – 44.54)	3989	47.63	(46.56 – 48.70)
Primary/junior high school	841	4.70	(4.39 – 5.01)	590	9.90	(9.17 – 10.68)	528	5.43	(5.00 – 5.90)	718	8.57	(7.99 – 9.19)
Subjective financial state												
Wealthy	1201	6.71	(6.35 – 7.08)	269	4.52	(4.01 – 5.07)	676	6.96	(6.46 – 7.48)	431	5.15	(4.69 – 5.64)

Nor poor not wealthy	7701	43.00	(42.27 – 43.72)	2175	36.51	(35.30 – 37.74)	4352	44.79	(43.80 – 45.78)	3253	38.84	(37.80 – 39.89)
Poor	9009	50.30	(49.57 – 51.03)	3513	58.97	(57.72 – 60.22)	4688	48.25	(47.26 – 49.24)	4691	56.01	(54.95 – 57.07)
Health insurance												
Employee insurance	13320	74.37	(73.72 – 75.00)	2778	46.63	(45.37 – 47.90)	6768	69.66	(68.74 – 70.57)	4419	52.76	(51.69 – 53.83)
National Health Insurance	4357	24.33	(23.70 – 24.96)	2958	49.66	(48.39 – 50.93)	2824	29.07	(28.17 – 29.97)	3686	44.01	(42.95 – 45.08)
Other	234	1.31	(1.15 – 1.48)	221	3.71	(3.25 – 4.21)	124	1.28	(1.07 – 1.51)	270	3.22	(2.86 – 3.62)
Employment status												
Employed	12383	69.14	(68.46 – 69.81)	1984	33.31	(32.12 – 34.51)	6176	63.57	(62.60 – 64.52)	3755	44.84	(43.77 – 45.90)
Self-employed	945	5.28	(4.96 – 5.61)	646	10.84	(10.07 – 11.65)	623	6.41	(5.94 – 6.91)	813	9.71	(9.09 – 10.36)
Employed(other)	1080	6.03	(5.69 – 6.39)	542	9.10	(8.39 – 9.85)	696	7.16	(6.66 – 7.69)	683	8.16	(7.58 – 8.76)
Unemployed	3503	19.56	(18.98 – 20.14)	2785	46.75	(45.49 – 48.02)	2221	22.86	(22.03 – 23.70)	3124	37.30	(36.27 – 38.34)
Kessler Psychological Distress Scale												
Normal(total score =< 4)	13610	75.99	(75.36 – 76.61)	4285	71.93	(70.78 – 73.06)	7522	77.42	(76.58 – 78.24)	6283	75.02	(74.09 – 75.94)
Mild illness (5 ≦ total score ≦ 12)	3701	20.66	(20.08 – 21.26)	1301	21.84	(20.80 – 22.90)	1930	19.86	(19.08 – 20.67)	1763	21.05	(20.19 – 21.93)
Severe illness (13 ≦ total score)	600	3.35	(3.09 – 3.62)	371	6.23	(5.64 – 6.86)	264	2.72	(2.41 – 3.05)	329	3.93	(3.53 – 4.36)
Constant visit to hospitals †												
Yes (constant visit)	8409	46.95	(46.22 – 47.68)	2651	44.50	(43.24 – 45.77)	5514	56.75	(55.76 – 57.73)	4188	50.01	(48.94 – 51.08)
No (no-constant visit)	9502	53.05	(52.32 – 53.78)	3306	55.50	(54.23 – 56.76)	4202	43.25	(42.27 – 44.24)	4187	49.99	(48.92 – 51.06)
Subjective health status												
Good	7307	40.80	(40.08 – 41.52)	2128	35.72	(34.51 – 36.95)	3720	38.29	(37.32 – 39.26)	2940	35.10	(34.09 – 36.13)
Normal	8877	49.56	(48.83 – 50.29)	3029	50.85	(49.58 – 52.12)	4958	51.03	(50.04 – 52.02)	4369	52.17	(51.10 – 53.24)
Bad	1727	9.64	(9.22 – 10.08)	800	13.43	(12.58 – 14.31)	1038	10.68	(10.08 – 11.31)	1066	12.73	(12.03 – 13.46)
Alcohol consumption												
Never or quit drinking	9498	53.03	(52.30 – 53.76)	3878	65.10	(63.88 – 66.30)	4805	49.45	(48.46 – 50.45)	4929	58.85	(57.80 – 59.90)
Social drinker/low-risk group (> 0 to ≤ 100 g/week)	4259	23.78	(23.16 – 24.41)	1061	17.81	(16.86 – 18.80)	2343	24.11	(23.27 – 24.97)	1606	19.18	(18.34 – 20.03)
Middle- risk drinking (> 100 to ≤ 350 g/week)	3519	19.65	(19.07 – 20.23)	837	14.05	(13.19 – 14.95)	2205	22.69	(21.87 – 23.54)	1536	18.34	(17.52 – 19.18)
High-risk drinking (> 350 g/week)	635	3.55	(3.28 – 3.82)	181	3.04	(2.63 – 3.50)	363	3.74	(3.37 – 4.13)	304	3.63	(3.25 – 4.05)
Smoking habit												

Never/ex-smoker	14812	82.70	(	82.14	–	83.25	)	4840	81.25	(	80.24	–	82.22	)	8103	83.40	(	82.65	–	84.13	)	6708	80.10	(	79.23	–	80.94	)
Current smoker	3099	17.30	(	16.75	–	17.86	)	1117	18.75	(	17.78	–	19.76	)	1613	16.60	(	15.87	–	17.35	)	1667	19.90	(	19.06	–	20.77	)

95% CI; 95% confidence interval, WG; the Washington Group Short Set

† No clear definition regarding the frequency of visit is provided in the questionnaire.

Table S2b. Participant characteristics stratified by participation or nonparticipation in colorectal and lung cancer screening.

	Colorectal cancer screening (n=18,132)						Gastric cancer screening (n=13,609)					
	Participants (n= 8,788)			Non participant (n= 9,344)			Participants (n=5,967)			Non participant (n=7,642)		
	Number	Percent	(95% CI)	Number	Percent	(95% CI)	Number	Percent	(95% CI)	Number	Percent	(95% CI)
Disability												
No-need any support or supervision	8686	98.84	(98.60 − 99.05)	9105	97.44	(97.11 – 97.75)	5886	98.64	(98.32 – 98.91)	7416	97.04	(96.64 – 97.40)
Need any support or supervision	102	1.16	(0.95 – 1.40)	239	2.56	(2.25 – 2.89)	81	1.36	(1.09 – 1.68)	226	2.96	(2.60 – 3.36)
WG sub-questions (vision)												
"No difficulty" or "some difficulty"	8505	96.78	(96.40 – 97.13)	9028	96.62	(96.24 – 96.97)	5762	96.56	(96.08 – 97.00)	7366	96.39	(95.95 – 96.79)
"A lot of difficulty" or "cannot do at all"	283	3.22	(2.87 – 3.60)	316	3.38	(3.03 – 3.76)	205	3.44	(3.00 – 3.92)	276	3.61	(3.21 – 4.05)
WG sub questions (hearing)												
"No difficulty" or "some difficulty"	8629	98.19	(97.90 – 98.45)	9143	97.85	(97.54 – 98.13)	5847	97.99	(97.61 – 98.32)	7476	97.83	(97.48 – 98.14)
"A lot of difficulty" or "cannot do at all"	159	1.81	(1.55 – 2.10)	201	2.15	(1.87 – 2.46)	120	2.01	(1.68 – 2.39)	166	2.17	(1.86 – 2.52)
WG sub questions (mobility)												
"No difficulty" or "some difficulty"	8547	97.26	(96.90 – 97.58)	8915	95.41	(94.97 – 95.82)	5777	96.82	(96.35 – 97.24)	7247	94.83	(94.32 – 95.31)
"A lot of difficulty" or "cannot do at all"	241	2.74	(2.42 – 3.10)	429	4.59	(4.18 – 5.03)	190	3.18	(2.76 – 3.65)	395	5.17	(4.69 – 5.68)
WG sub questions (remembering or concentrating)												
"No difficulty" or "some difficulty"	8647	98.40	(98.12 – 98.64)	9109	97.49	(97.15 – 97.79)	5863	98.26	(97.90 – 98.57)	7463	97.66	(97.30 – 97.98)
"A lot of difficulty" or "cannot do at all"	141	1.60	(1.36 – 1.88)	235	2.51	(2.21 – 2.85)	104	1.74	(1.43 – 2.10)	179	2.34	(2.02 – 2.70)
WG sub questions (self-care)												

"No difficulty" or "some difficulty"	8676	98.73	(98.47 – 98.94)	9151	97.93	(97.63 – 98.21)	5893	98.76	(98.45 – 99.02)	7484	97.93	(97.60 – 98.23)
"A lot of difficulty" or "cannot do at all"	112	1.27	(1.06 – 1.53)	193	2.07	(1.79 – 2.37)	74	1.24	(0.98 – 1.55)	158	2.07	(1.77 – 2.40)
WG sub questions (communication)												
"No difficulty" or "some difficulty"	8678	98.75	(98.50 – 98.96)	9138	97.80	(97.48 – 98.08)	5897	98.83	(98.53 – 99.08)	7497	98.10	(97.78 – 98.39)
"A lot of difficulty" or "cannot do at all"	110	1.25	(1.04 – 1.50)	206	2.20	(1.92 – 2.52)	70	1.17	(0.92 – 1.47)	145	1.90	(1.61 – 2.22)
Numbers of difficulty defined by WG												
0	8204	93.35	(92.82 – 93.86)	8530	91.29	(90.70 – 91.85)	5515	92.43	(91.73 – 93.08)	6906	90.37	(89.69 – 91.02)
1	418	4.76	(4.33 – 5.22)	520	5.57	(5.11 – 6.04)	331	5.55	(4.99 – 6.15)	487	6.37	(5.84 – 6.94)
2 and more	166	1.89	(1.62 – 2.19)	294	3.15	(2.81 – 3.51)	121	2.03	(1.69 – 2.41)	249	3.26	(2.88 – 3.67)
Sex												
Male	4567	51.97	(50.92 – 53.01)	4189	44.83	(43.82 – 45.84)	3226	54.06	(52.80 – 55.33)	3282	42.95	(41.84 – 44.06)
Female	4221	48.03	(46.99 – 49.08)	5155	55.17	(54.16 – 56.18)	2741	45.94	(44.67 – 47.20)	4360	57.05	(55.94 – 58.16)
Age (years)												
65–74	2737	31.14	(30.18 – 32.12)	3256	34.85	(33.88 – 35.82)	-	-	(- – -)	-	-	(- – -)
40–64	6051	68.86	(67.88 – 69.82)	6088	65.15	(64.18 – 66.12)	-	-	(- – -)	-	-	(- – -)
65–74	-	-	(- – -)	-	-	(- – -)	2393	40.10	(38.87 – 41.35)	3569	46.70	(45.59 – 47.82)
50–64	-	-	(- – -)	-	-	(- – -)	3574	59.90	(58.65 – 61.13)	4073	53.30	(52.18 – 54.41)
Marital status												
Married	6991	79.55	(78.70 – 80.39)	6567	70.28	(69.35 – 71.20)	4799	80.43	(79.40 – 81.42)	5598	73.25	(72.25 – 74.24)
Single	953	10.84	(10.21 – 11.51)	1574	16.85	(16.10 – 17.61)	527	8.83	(8.13 – 9.57)	933	12.21	(11.49 – 12.96)
Divorced/widowed	844	9.60	(9.00 – 10.23)	1203	12.87	(12.21 – 13.57)	641	10.74	(9.98 – 11.55)	1111	14.54	(13.76 – 15.34)
Educational qualification												
Vocational school/junior college/community(technical) college/university/post graduate school	4557	51.85	(50.81 – 52.90)	4081	43.68	(42.67 – 44.68)	2879	48.25	(46.98 – 49.52)	3034	39.70	(38.61 – 40.80)
High school	3791	43.14	(42.11 – 44.18)	4447	47.59	(46.58 – 48.61)	2750	46.09	(44.82 – 47.35)	3885	50.84	(49.72 – 51.96)
Primary/junior high school	440	5.01	(4.57 – 5.48)	816	8.73	(8.17 – 9.32)	338	5.66	(5.10 – 6.27)	723	9.46	(8.82 – 10.13)
Subjective financial state												

Wealthy	635	7.23	(6.70 – 7.78)	476	5.09	(4.66 – 5.55)	441	7.39	(6.75 – 8.08)	409	5.35	(4.86 – 5.87)
Nor poor not wealthy	4027	45.82	(44.78 – 46.87)	3594	38.46	(37.48 – 39.45)	2769	46.41	(45.14 – 47.67)	3039	39.77	(38.67 – 40.87)
Poor	4126	46.95	(45.91 – 47.99)	5274	56.44	(55.44 – 57.45)	2757	46.20	(44.94 – 47.47)	4194	54.88	(53.76 – 55.99)
Health insurance												
Employee insurance	6117	69.61	(68.64 – 70.56)	5086	54.43	(53.42 – 55.44)	3852	64.56	(63.33 – 65.76)	3535	46.26	(45.14 – 47.38)
National Health Insurance	2543	28.94	(28.00 – 29.89)	3991	42.71	(41.71 – 43.72)	2027	33.97	(32.78 – 35.18)	3852	50.41	(49.28 – 51.53)
Other	128	1.46	(1.22 – 1.72)	267	2.86	(2.53 – 3.21)	88	1.47	(1.19 – 1.80)	255	3.34	(2.95 – 3.76)
Employment status												
Employed	5495	62.53	(61.51 – 63.54)	4447	47.59	(46.58 – 48.61)	3418	57.28	(56.02 – 58.53)	3084	40.36	(39.26 – 41.46)
Self-employed	586	6.67	(6.16 – 7.20)	855	9.15	(8.58 – 9.75)	435	7.29	(6.65 – 7.97)	754	9.87	(9.21 – 10.55)
Employed(other)	647	7.36	(6.83 – 7.92)	734	7.86	(7.32 – 8.41)	503	8.43	(7.74 – 9.15)	586	7.67	(7.09 – 8.28)
Unemployed	2060	23.44	(22.56 – 24.34)	3308	35.40	(34.44 – 36.38)	1611	27.00	(25.88 – 28.14)	3218	42.11	(41.01 – 43.22)
Kessler Psychological Distress Scale												
Normal (total score =< 4)	6796	77.33	(76.45 – 78.20)	7041	75.35	(74.47 – 76.22)	4696	78.70	(77.65 – 79.72)	5869	76.80	(75.84 – 77.74)
Mild illness (5 ≦ total score ≦ 12)	1759	20.02	(19.19 – 20.86)	1943	20.79	(19.98 – 21.63)	1137	19.05	(18.07 – 20.07)	1536	20.10	(19.21 – 21.01)
Severe illness (13 ≦ total score)	233	2.65	(2.33 – 3.00)	360	3.85	(3.48 – 4.26)	134	2.25	(1.89 – 2.65)	237	3.10	(2.73 – 3.51)
Constant visit to hospitals †												
Yes (constant visit)	4993	56.82	(55.78 – 57.85)	4732	50.64	(49.63 – 51.66)	3819	64.00	(62.78 – 65.21)	4457	58.32	(57.21 – 59.42)
No (no-constant visit)	3795	43.18	(42.15 – 44.22)	4612	49.36	(48.34 – 50.37)	2148	36.00	(34.79 – 37.22)	3185	41.68	(40.58 – 42.79)
Subjective health status												
Good	3416	38.87	(37.86 – 39.89)	3252	34.80	(33.84 – 35.77)	2199	36.85	(35.64 – 38.08)	2534	33.16	(32.11 – 34.22)
Normal	4438	50.50	(49.46 – 51.55)	4915	52.60	(51.59 – 53.61)	3062	51.32	(50.05 – 52.58)	4067	53.22	(52.10 – 54.34)
Bad	934	10.63	(10.00 – 11.29)	1177	12.60	(11.94 – 13.28)	706	11.83	(11.03 – 12.67)	1041	13.62	(12.87 – 14.40)
Alcohol consumption												
Never or quit drinking	4308	49.02	(47.98 – 50.07)	5450	58.33	(57.32 – 59.32)	2839	47.58	(46.31 – 48.85)	4516	59.09	(57.99 – 60.19)
Social drinker/low-risk group (> 0 to ≤ 100 g/week)	2152	24.49	(23.60 – 25.40)	1809	19.36	(18.57 – 20.17)	1458	24.43	(23.36 – 25.54)	1470	19.24	(18.36 – 20.13)
Middle- risk drinking (> 100 to ≤ 350 g/week)	2014	22.92	(22.05 – 23.81)	1731	18.53	(17.75 – 19.32)	1483	24.85	(23.77 – 25.96)	1387	18.15	(17.30 – 19.03)
High-risk drinking (> 350 g/week)	314	3.57	(3.20 – 3.98)	354	3.79	(3.42 – 4.19)	187	3.13	(2.71 – 3.60)	269	3.52	(3.12 – 3.95)

Smoking habit																												
Never/ex-smoker	7418	84.41	(	83.64	–	85.16	)	7433	79.55	(	78.72	–	80.36	)	5054	84.70	(	83.77	–	85.60	)	6222	81.42	(	80.53	–	82.28	)
Current smoker	1370	15.59	(	14.84	–	16.36	)	1911	20.45	(	19.64	–	21.28	)	913	15.30	(	14.40	–	16.23	)	1420	18.58	(	17.72	–	19.47	)

95% CI; 95% confidence interval, WG; the Washington Group Short Set

† No clear definition regarding the frequency of visit is provided in the questionnaire.

Table S2c. Participant characteristics stratified by participation or nonparticipation in cervical and breast cancer screening.

	Cervical cancer screening (n=12,165)						Breast cancer screening (n=9,364)					
	Participants(n=5,318)			Non participant(n=6,847)			Participants (n=4,402)			Non participant (n=4,962)		
	Number	Percent	(95% CI)	Number	Percent	(95% CI)	Number	Percent	(95% CI)	Number	Percent	(95% CI)
Disability												
No-need any support or supervision	5270	99.10	(98.82 – 99.33)	6683	97.60	(97.22 – 97.95)	4367	99.20	(98.91 – 99.44)	4833	97.40	(96.93 – 97.82)
Need any support or supervision	48	0.90	(0.67 – 1.18)	164	2.40	(2.05 – 2.78)	35	0.80	(0.56 – 1.09)	129	2.60	(2.18 – 3.07)
WG sub-questions (vision)												
"No difficulty" or "some difficulty"	5167	97.16	(96.69 – 97.58)	6643	97.02	(96.60 – 97.40)	4278	97.18	(96.66 – 97.64)	4804	96.82	(96.30 – 97.28)
"A lot of difficulty" or "cannot do at all"	151	2.84	(2.42 – 3.31)	204	2.98	(2.60 – 3.40)	124	2.82	(2.36 – 3.34)	158	3.18	(2.72 – 3.70)
WG sub questions (hearing)												
"No difficulty" or "some difficulty"	5229	98.33	(97.95 – 98.65)	6715	98.07	(97.73 – 98.38)	4330	98.36	(97.96 – 98.71)	4863	98.00	(97.59 – 98.37)
"A lot of difficulty" or "cannot do at all"	89	1.67	(1.35 – 2.05)	132	1.93	(1.62 – 2.27)	72	1.64	(1.29 – 2.04)	99	2.00	(1.63 – 2.41)
WG sub questions (mobility)												
"No difficulty" or "some difficulty"	5172	97.25	(96.79 – 97.67)	6567	95.91	(95.42 – 96.36)	4271	97.02	(96.49 – 97.50)	4734	95.41	(94.80 – 95.96)
"A lot of difficulty" or "cannot do at all"	146	2.75	(2.33 – 3.21)	280	4.09	(3.64 – 4.58)	131	2.98	(2.50 – 3.51)	228	4.59	(4.04 – 5.20)
WG sub questions (remembering or concentrating)												
"No difficulty" or "some difficulty"	5217	98.10	(97.71 – 98.44)	6695	97.78	(97.41 – 98.11)	4338	98.55	(98.16 – 98.87)	4858	97.90	(97.48 – 98.28)
"A lot of difficulty" or "cannot do at all"	101	1.90	(1.56 – 2.29)	152	2.22	(1.89 – 2.59)	64	1.45	(1.13 – 1.84)	104	2.10	(1.72 – 2.52)
WG sub questions (self-care)												

“No difficulty” or “some difficulty”	5237	98.48	(98.12 – 98.78)	6709	97.98	(97.63 – 98.30)	4345	98.71	(98.34 – 99.01)	4869	98.13	(97.72 – 98.48)
“A lot of difficulty” or “cannot do at all”	81	1.52	(1.22 – 1.88)	138	2.02	(1.70 – 2.37)	57	1.29	(0.99 – 1.66)	93	1.87	(1.52 – 2.28)
WG sub questions (communication)												
“No difficulty” or “some difficulty”	5231	98.36	(98.00 – 98.68)	6702	97.88	(97.52 – 98.20)	4347	98.75	(98.39 – 99.05)	4874	98.23	(97.83 – 98.57)
“A lot of difficulty” or “cannot do at all”	87	1.64	(1.32 – 2.00)	145	2.12	(1.80 – 2.48)	55	1.25	(0.95 – 1.61)	88	1.77	(1.43 – 2.17)
Numbers of difficulty defined by WG												
0	4999	94.00	(93.34 – 94.62)	6311	92.17	(91.52 – 92.79)	4136	93.96	(93.22 – 94.63)	4534	91.37	(90.57 – 92.13)
1	214	4.02	(3.52 – 4.58)	351	5.13	(4.62 – 5.67)	188	4.27	(3.70 – 4.90)	292	5.88	(5.26 – 6.57)
2 and more	105	1.97	(1.63 – 2.37)	185	2.70	(2.34 – 3.11)	78	1.77	(1.41 – 2.19)	136	2.74	(2.31 – 3.22)
Sex												
Male	0	0.00	(–)	0	0.00	(–)	0	0.00	(–)	0	0.00	(–)
Female	5318	100.00	(–)	6847	100.00	(–)	4402	100.00	(–)	4962	100.00	(–)
Age (years)												
65–74	947	17.81	(16.80 – 18.85)	2115	30.89	(29.80 – 31.99)	1135	25.78	(24.51 – 27.09)	1942	39.14	(37.79 – 40.50)
40–64	3161	59.44	(58.12 – 60.75)	3118	45.54	(44.36 – 46.72)	3267	74.22	(72.91 – 75.49)	3020	60.86	(59.50 – 62.21)
20–39	1210	22.75	(21.64 – 23.89)	1614	23.57	(22.58 – 24.59)	0	0.00	(–)	0	0.00	(–)
Marital status												
Married	3993	75.08	(73.91 – 76.23)	4163	60.80	(59.64 – 61.95)	3420	77.69	(76.44 – 78.90)	3506	70.66	(69.38 – 71.91)
Single	739	13.90	(12.99 – 14.85)	1748	25.53	(24.51 – 26.57)	406	9.22	(8.40 – 10.10)	602	12.13	(11.25 – 13.06)
Divorced/widowed	586	11.02	(10.20 – 11.88)	936	13.67	(12.87 – 14.50)	576	13.08	(12.11 – 14.11)	854	17.21	(16.18 – 18.28)
Educational qualification												
Vocational school/junior college/community(technical) college/university/post graduate school	3125	58.76	(57.44 – 60.08)	3296	48.14	(46.96 – 49.32)	2377	54.00	(52.52 – 55.47)	2087	42.06	(40.69 – 43.44)
High school	2021	38.00	(36.71 – 39.31)	3081	45.00	(43.82 – 46.18)	1851	42.05	(40.60 – 43.51)	2482	50.02	(48.63 – 51.41)
Primary/junior high school	172	3.23	(2.78 – 3.74)	470	6.86	(6.28 – 7.48)	174	3.95	(3.41 – 4.56)	393	7.92	(7.19 – 8.70)
Subjective financial state												
Wealthy	376	7.07	(6.40 – 7.78)	361	5.27	(4.76 – 5.82)	311	7.06	(6.34 – 7.85)	246	4.96	(4.38 – 5.59)

Nor poor not wealthy	2357	44.32	(42.99 – 45.66)	2666	38.94	(37.79 – 40.10)	1990	45.21	(43.74 – 46.68)	1929	38.88	(37.53 – 40.24)
Poor	2585	48.61	(47.27 – 49.95)	3820	55.79	(54.61 – 56.96)	2101	47.73	(46.25 – 49.20)	2787	56.17	(54.78 – 57.54)
Health insurance												
Employee insurance	4057	76.29	(75.13 – 77.42)	4093	59.78	(58.61 – 60.94)	3089	70.17	(68.81 – 71.51)	2618	52.76	(51.37 – 54.15)
National Health Insurance	1214	22.83	(21.72 – 23.97)	2599	37.96	(36.81 – 39.11)	1276	28.99	(27.66 – 30.34)	2196	44.26	(42.88 – 45.64)
Other	47	0.88	(0.66 – 1.16)	155	2.26	(1.93 – 2.64)	37	0.84	(0.60 – 1.14)	148	2.98	(2.54 – 3.48)
Employment status												
Employed	3407	64.07	(62.77 – 65.35)	3481	50.84	(49.66 – 52.02)	2608	59.25	(57.79 – 60.69)	2177	43.87	(42.50 – 45.26)
Self-employed	160	3.01	(2.57 – 3.49)	208	3.04	(2.65 – 3.46)	139	3.16	(2.67 – 3.71)	188	3.79	(3.28 – 4.35)
Employed(other)	346	6.51	(5.87 – 7.19)	480	7.01	(6.42 – 7.63)	304	6.91	(6.19 – 7.68)	417	8.40	(7.66 – 9.20)
Unemployed	1405	26.42	(25.25 – 27.62)	2678	39.11	(37.96 – 40.27)	1351	30.69	(29.34 – 32.07)	2180	43.93	(42.56 – 45.32)
Kessler Psychological Distress Scale												
Normal(total score =< 4)	3832	72.06	(70.84 – 73.25)	4979	72.72	(71.65 – 73.76)	3256	73.97	(72.65 – 75.25)	3671	73.98	(72.75 – 75.19)
Mild illness (5 ≦ total score ≦ 12)	1252	23.54	(22.42 – 24.70)	1541	22.51	(21.53 – 23.51)	1020	23.17	(21.94 – 24.44)	1080	21.77	(20.63 – 22.93)
Severe illness (13 ≦ total score)	234	4.40	(3.87 – 4.98)	327	4.78	(4.29 – 5.30)	126	2.86	(2.40 – 3.39)	211	4.25	(3.72 – 4.84)
Constant visit to hospitals †												
Yes (constant visit)	2546	47.88	(46.53 – 49.22)	3177	46.40	(45.22 – 47.58)	2401	54.54	(53.07 – 56.01)	2607	52.54	(51.15 – 53.93)
No (no-constant visit)	2772	52.12	(50.78 – 53.47)	3670	53.60	(52.42 – 54.78)	2001	45.46	(43.99 – 46.93)	2355	47.46	(46.07 – 48.85)
Subjective health status												
Good	2240	42.12	(40.80 – 43.45)	2532	36.98	(35.84 – 38.13)	1765	40.10	(38.65 – 41.55)	1683	33.92	(32.61 – 35.24)
Normal	2523	47.44	(46.10 – 48.79)	3567	52.10	(50.91 – 53.28)	2182	49.57	(48.09 – 51.05)	2663	53.67	(52.28 – 55.05)
Bad	555	10.44	(9.64 – 11.28)	748	10.92	(10.20 – 11.68)	455	10.34	(9.46 – 11.26)	616	12.41	(11.52 – 13.35)
Alcohol consumption												
Never or quit drinking	3490	65.63	(64.34 – 66.89)	4887	71.37	(70.29 – 72.44)	2831	64.31	(62.89 – 65.72)	3523	71.00	(69.73 – 72.25)
Social drinker/low-risk group (> 0 to ≤ 100 g/week)	1266	23.81	(22.68 – 24.96)	1318	19.25	(18.33 – 20.20)	1071	24.33	(23.08 – 25.61)	930	18.74	(17.68 – 19.85)
Middle- risk drinking (> 100 to ≤ 350 g/week)	498	9.36	(8.60 – 10.17)	550	8.03	(7.41 – 8.69)	453	10.29	(9.42 – 11.21)	429	8.65	(7.89 – 9.45)
High-risk drinking (> 350 g/week)	64	1.20	(0.94 – 1.52)	92	1.34	(1.09 – 1.64)	47	1.07	(0.80 – 1.40)	80	1.61	(1.29 – 1.99)
Smoking habit												

Never/ex-smoker	5018	94.36	(	93.71	–	94.95	)	6223	90.89	(	90.19	–	91.55	)	4172	94.78	(	94.09	–	95.40	)	4464	89.96	(	89.10	–	90.78	)
Current smoker	300	5.64	(	5.05	–	6.29	)	624	9.11	(	8.45	–	9.81	)	230	5.22	(	4.60	–	5.91	)	498	10.04	(	9.22	–	10.90	)

95% CI; 95% confidence interval, WG; the Washington Group Short Set

† No clear definition regarding the frequency of visit is provided in the questionnaire.
