# Supplementary material for: Functional Limitations and Use of General Health Examination and Cancer Screening Among People with Disabilities Who Need Support from Others: Secondary Data Analysis of the 2022 Comprehensive Survey of Living Conditions in Japan
Source: Int J Environ Res Public Health. 2025 Mar 24;22(4):484. doi: 10.3390/ijerph22040484 (PMC12026762; doi:10.3390/ijerph22040484)
Supplement: Supplementary file 1 [file ijerph-22-00484-s001.zip › Suppl3.pdf]

Table S3a Unadjusted and adjusted odds ratio for non-participation in the general health examination and lung cancer screening.

	General health examination (n=23,868)				Lung cancer screening(n=18,091)		
	Unadjusted OR		Adjusted OR		Unadjusted OR	Adjusted OR	
	OR	(95%CI)	OR(95%CI)	P	OR(95%CI)	OR(95%CI)	P
Disability							
No-need any support or supervision	1.000	(Ref.)	1.000 (Ref.)	Ref.	1.000 (Ref.)	1.000 (Ref.)	Ref.
Need any support or supervision	2.986	(2.473 – 3.606)	1.340 (1.085 – 1.655)	0.007	2.264 (1.805 – 2.839)	1.436 (1.127 – 1.830)	0.003
Sex							
Male	1.000	(Ref.)	1.000 (Ref.)	Ref.	1.000 (Ref.)	1.000 (Ref.)	Ref.
Female	1.476	(1.391 – 1.566)	1.434 (1.333 – 1.543)	<0.001	1.350 (1.273 – 1.432)	1.320 (1.231 – 1.415)	<0.001
Age (years)							
65–74	1.000	(Ref.)	1.000 (Ref.)	Ref.	1.000 (Ref.)	1.000 (Ref.)	Ref.
40–64	0.595	(0.555 – 0.638)	1.461 (1.332 – 1.603)	<0.001	0.835 (0.785 – 0.888)	1.303 (1.198 – 1.418)	<0.001
20–39	0.802	(0.740 – 0.869)	2.037 (1.804 – 2.300)	<0.001	NA (NA)	NA (NA)	NA
Marital status							
Married	1.000	(Ref.)	1.000 (Ref.)	Ref.	1.000 (Ref.)	1.000 (Ref.)	Ref.
Single	1.437	(1.343 – 1.538)	1.359 (1.248 – 1.481)	<0.001	1.581 (1.452 – 1.722)	1.475 (1.344 – 1.618)	<0.001
Divorced/widowed	1.371	(1.241 – 1.514)	1.099 (0.984 – 1.229)	0.095	1.374 (1.251 – 1.508)	1.165 (1.055 – 1.287)	0.003
Constant visit to hospitals †							
Yes (constant visit)	1.000	(Ref.)	1.000 (Ref.)	Ref.	1.000 (Ref.)	1.000 (Ref.)	Ref.
No (no-constant visit)	1.104	(1.040 – 1.171)	1.538 (1.431 – 1.654)	<0.001	1.312 (1.237 – 1.391)	1.575 (1.475 – 1.682)	<0.001
Subjective health status							
Good	1.000	(Ref.)	1.000 (Ref.)	Ref.	1.000 (Ref.)	1.000 (Ref.)	Ref.
Normal	1.172	(1.100 – 1.248)	1.115 (1.038 – 1.197)	0.003	1.115 (1.047 – 1.188)	1.072 (1.002 – 1.147)	0.044
Bad	1.591	(1.444 – 1.752)	1.215 (1.077 – 1.372)	0.002	1.299 (1.178 – 1.434)	1.137 (1.014 – 1.276)	0.028
Alcohol consumption							
Never or quit drinking	1.000	(Ref.)	1.000 (Ref.)	Ref.	1.000 (Ref.)	1.000 (Ref.)	Ref.
Social drinker/low-risk group (> 0 to ≤ 100 g/week)	0.610	(0.565 – 0.659)	0.753 (0.692 – 0.819)	<0.001	0.668 (0.620 – 0.720)	0.772 (0.713 – 0.835)	<0.001
Middle- risk drinking (> 100 to ≤ 350 g/week)	0.583	(0.536 – 0.634)	0.775 (0.704 – 0.854)	<0.001	0.679 (0.629 – 0.733)	0.823 (0.756 – 0.896)	<0.001
High-risk drinking (> 350 g/week)	0.698	(0.589 – 0.827)	0.898 (0.744 – 1.084)	0.264	0.816 (0.697 – 0.956)	0.952 (0.805 – 1.126)	0.563
Smoking habit							
Never/ex-smoker	1.000	(Ref.)	1.000 (Ref.)	Ref.	1.000 (Ref.)	1.000 (Ref.)	Ref.
Current smoker	1.103	(1.023 – 1.190)	1.290 (1.179 – 1.411)	<0.001	1.248 (1.157 – 1.347)	1.335 (1.227 – 1.453)	<0.001
Educational qualification							

Vocational school/junior college/community(technical) college/university/post graduate school	1.000	(Ref.)	1.000	(Ref.)	Ref.	1.000	(Ref.)	1.000	(Ref.)	Ref.
High school	1.334	(1.254 – 1.419)	1.171	(1.093 – 1.255)	<0.001	1.274	(1.198 – 1.353)	1.140	(1.068 – 1.216)	<0.001
Primary/junior high school	2.552	(2.278 – 2.860)	1.737	(1.525 – 1.978)	<0.001	1.837	(1.629 – 2.072)	1.382	(1.213 – 1.574)	<0.001
Subjective financial state										
Wealthy	1.000	(Ref.)	1.000	(Ref.)	Ref.	1.000	(Ref.)	1.000	(Ref.)	Ref.
Nor poor not wealthy	1.261	(1.096 – 1.451)	1.218	(1.047 – 1.415)	0.010	1.172	(1.030 – 1.334)	1.114	(0.974 – 1.275)	0.116
Poor	1.741	(1.517 – 1.998)	1.530	(1.318 – 1.776)	<0.001	1.569	(1.382 – 1.783)	1.386	(1.212 – 1.586)	<0.001
Kessler Psychological Distress Scale										
Normal (total score =< 4)	1.000	(Ref.)	1.000	(Ref.)	Ref.	1.000	(Ref.)	1.000	(Ref.)	Ref.
Mild illness (5 ≦ total score ≦ 12)	1.117	(1.039 – 1.200)	1.011	(0.931 – 1.098)	0.796	1.094	(1.017 – 1.176)	1.010	(0.932 – 1.093)	0.813
Severe illness (13 ≦ total score)	1.964	(1.718 – 2.245)	1.442	(1.228 – 1.694)	<0.001	1.492	(1.265 – 1.760)	1.180	(0.983 – 1.416)	0.076
Health insurance										
Employee insurance	1.000	(Ref.)	1.000	(Ref.)	Ref.	1.000	(Ref.)	1.000	(Ref.)	Ref.
National Health Insurance	3.255	(3.059 – 3.464)	1.889	(1.731 – 2.062)	<0.001	1.999	(1.879 – 2.127)	1.503	(1.377 – 1.639)	<0.001
Other	4.528	(3.751 – 5.467)	2.450	(1.980 – 3.031)	<0.001	3.335	(2.687 – 4.139)	2.268	(1.798 – 2.860)	<0.001
Employment status										
Employed	1.000	(Ref.)	1.000	(Ref.)	Ref.	1.000	(Ref.)	1.000	(Ref.)	Ref.
Self-employed	4.267	(3.819 – 4.766)	3.981	(3.491 – 4.540)	<0.001	2.146	(1.919 – 2.401)	1.951	(1.712 – 2.222)	<0.001
Employed(other)	3.132	(2.796 – 3.509)	3.251	(2.873 – 3.679)	<0.001	1.614	(1.441 – 1.807)	1.634	(1.448 – 1.844)	<0.001
Unemployed	4.962	(4.633 – 5.315)	4.485	(4.109 – 4.896)	<0.001	2.313	(2.162 – 2.476)	2.080	(1.902 – 2.274)	<0.001
Model chi square test			P<0.001					P<0.001		
Hosmer-Lemeshow test			P=0.145					P=0.391		

Note: OR, odds ratio; CI, confidence interval; Ref., reference; NA, not available

† No clear definition regarding the frequency of visit is provided in the questionnaire.

Table S3b Unadjusted and adjusted odds ratio for non-participation in the colorectal and lung cancer screening.

	Colorectal cancer screening (n=18,132)					Gastric cancer screening (n=13,609)				
	Unadjusted OR		Adjusted OR			Unadjusted OR		Adjusted OR		
	OR	(95%CI)	OR	(95%CI)	P	OR	(95%CI)	OR	(95%CI)	P
Disability										
No-need any support or supervision	1.000	(Ref.)	1.000	(Ref.)	Ref.	1.000	(Ref.)	1.000	(Ref.)	Ref.
Need any support or supervision	2.235	(1.770 – 2.824)	1.515	(1.181 – 1.943)	0.001	2.214	(1.714 – 2.861)	1.535	(1.168 – 2.016)	0.002
Sex										
Male	1.000	(Ref.)	1.000	(Ref.)	Ref.	1.000	(Ref.)	1.000	(Ref.)	Ref.
Female	1.331	(1.256 – 1.412)	1.344	(1.254 – 1.440)	<0.001	1.564	(1.461 – 1.674)	1.520	(1.401 – 1.650)	<0.001
Age (years)										
65–74	1.000	(Ref.)	1.000	(Ref.)	Ref.	1.000	(Ref.)	1.000	(Ref.)	Ref.
40–64	0.846	(0.795 – 0.900)	1.204	(1.107 – 1.309)	<0.001	NA	(NA)	NA	(NA)	NA
50–64	NA	(NA)	NA	(NA)	NA	0.764	(0.713 – 0.818)	1.128	(1.031 – 1.234)	0.009
Marital status				(–)					(–)	
Married	1.000	(Ref.)	1.000	(Ref.)	Ref.	1.000	(Ref.)	1.000	(Ref.)	Ref.
Single	1.758	(1.611 – 1.918)	1.648	(1.501 – 1.810)	<0.001	1.518	(1.355 – 1.700)	1.390	(1.231 – 1.570)	<0.001
Divorced/widowed	1.517	(1.381 – 1.667)	1.266	(1.146 – 1.399)	<0.001	1.486	(1.338 – 1.650)	1.203	(1.076 – 1.344)	0.001
Constant visit to hospitals †										
Yes (constant visit)	1.000	(Ref.)	1.000	(Ref.)	Ref.	1.000	(Ref.)	1.000	(Ref.)	Ref.
No (no-constant visit)	1.282	(1.209 – 1.360)	1.506	(1.410 – 1.607)	<0.001	1.271	(1.185 – 1.362)	1.466	(1.358 – 1.583)	<0.001
Subjective health status										
Good	1.000	(Ref.)	1.000	(Ref.)	Ref.	1.000	(Ref.)	1.000	(Ref.)	Ref.
Normal	1.163	(1.092 – 1.239)	1.113	(1.041 – 1.190)	0.002	1.153	(1.071 – 1.241)	1.097	(1.014 – 1.188)	0.021
Bad	1.324	(1.200 – 1.461)	1.164	(1.039 – 1.305)	0.009	1.280	(1.145 – 1.430)	1.114	(0.979 – 1.268)	0.101
Alcohol consumption										
Never or quit drinking	1.000	(Ref.)	1.000	(Ref.)	Ref.	1.000	(Ref.)	1.000	(Ref.)	Ref.
Social drinker/low-risk group (> 0 to ≤ 100 g/week)	0.664	(0.617 – 0.716)	0.768	(0.711 – 0.830)	<0.001	0.634	(0.581 – 0.691)	0.749	(0.684 – 0.820)	<0.001
Middle- risk drinking (> 100 to ≤ 350 g/week)	0.679	(0.630 – 0.733)	0.816	(0.750 – 0.888)	<0.001	0.588	(0.539 – 0.641)	0.755	(0.685 – 0.833)	<0.001
High-risk drinking (> 350 g/week)	0.891	(0.762 – 1.043)	1.018	(0.861 – 1.202)	0.838	0.904	(0.746 – 1.096)	1.148	(0.935 – 1.408)	0.187
Smoking habit										
Never/ex-smoker	1.000	(Ref.)	1.000	(Ref.)	Ref.	1.000	(Ref.)	1.000	(Ref.)	Ref.
Current smoker	1.392	(1.290 – 1.503)	1.475	(1.355 – 1.605)	<0.001	1.263	(1.153 – 1.384)	1.426	(1.289 – 1.577)	<0.001
Educational qualification										

Vocational school/junior college/community(technical) college/university/post graduate school	1.000 (Ref.)	1.000 (Ref.)	Ref.	1.000 (Ref.)	1.000 (Ref.)	Ref.
High school	1.310 (1.233 – 1.392)	1.156 (1.084 – 1.233)	<0.001	1.341 (1.249 – 1.439)	1.172 (1.087 – 1.263)	<0.001
Primary/junior high school	2.071 (1.830 – 2.343)	1.561 (1.368 – 1.783)	<0.001	2.030 (1.767 – 2.332)	1.471 (1.267 – 1.708)	<0.001
Subjective financial state						
Wealthy	1.000 (Ref.)	1.000 (Ref.)	Ref.	1.000 (Ref.)	1.000 (Ref.)	Ref.
Nor poor not wealthy	1.191 (1.049 – 1.352)	1.102 (0.965 – 1.257)	0.151	1.183 (1.025 – 1.367)	1.065 (0.915 – 1.238)	0.416
Poor	1.705 (1.504 – 1.933)	1.466 (1.285 – 1.673)	<0.001	1.640 (1.422 – 1.892)	1.363 (1.172 – 1.586)	<0.001
Kessler Psychological Distress Scale						
Normal (total score =< 4)	1.000 (Ref.)	1.000 (Ref.)	Ref.	1.000 (Ref.)	1.000 (Ref.)	Ref.
Mild illness (5 ≦ total score ≦ 12)	1.066 (0.992 – 1.146)	0.967 (0.893 – 1.046)	0.401	1.081 (0.992 – 1.178)	0.978 (0.890 – 1.075)	0.649
Severe illness (13 ≦ total score)	1.491 (1.261 – 1.764)	1.146 (0.953 – 1.377)	0.148	1.415 (1.141 – 1.755)	1.096 (0.867 – 1.386)	0.445
Health insurance						
Employee insurance	1.000 (Ref.)	1.000 (Ref.)	Ref.	1.000 (Ref.)	1.000 (Ref.)	Ref.
National Health Insurance	1.888 (1.774 – 2.008)	1.496 (1.371 – 1.633)	<0.001	2.071 (1.930 – 2.222)	1.581 (1.435 – 1.741)	<0.001
Other	2.509 (2.026 – 3.107)	1.652 (1.312 – 2.081)	<0.001	3.158 (2.468 – 4.041)	2.103 (1.616 – 2.738)	<0.001
Employment status						
Employed	1.000 (Ref.)	1.000 (Ref.)	Ref.	1.000 (Ref.)	1.000 (Ref.)	Ref.
Self-employed	1.803 (1.611 – 2.017)	1.576 (1.383 – 1.797)	<0.001	1.921 (1.691 – 2.183)	1.640 (1.415 – 1.900)	<0.001
Employed(other)	1.402 (1.252 – 1.569)	1.416 (1.255 – 1.597)	<0.001	1.291 (1.135 – 1.469)	1.225 (1.067 – 1.407)	0.004
Unemployed	1.984 (1.854 – 2.123)	1.708 (1.562 – 1.867)	<0.001	2.214 (2.050 – 2.391)	1.690 (1.529 – 1.867)	<0.001
Model chi square test		P<0.001			P<0.001	
Hosmer-Lemeshow test		P=0.085			P=0.631	

Note: OR, odds ratio; CI, confidence interval; Ref., reference; NA, not available

† No clear definition regarding the frequency of visit is provided in the questionnaire.

Table S3c Unadjusted and adjusted odds ratio for non-participation in the cervical and breast cancer screening.

	Cervical cancer screening (n=12,165)					Breast cancer screening (n=9,364)				
	Unadjusted OR		Adjusted OR			Unadjusted OR		Adjusted OR		
	OR	(95%CI)	OR	(95%CI)	P	OR	(95%CI)	OR	(95%CI)	P
Disability										
No-need any support or supervision	1.000	(Ref.)	1.000	(Ref.)	Ref.	1.000	(Ref.)	1.000	(Ref.)	Ref.
Need any support or supervision	2.694	(1.949 – 3.724)	1.684	(1.193 – 2.375)	0.003	3.330	(2.287 – 4.849)	2.100	(1.415 – 3.116)	<0.001
Sex										
Male	NA	(NA)	NA	(NA)	NA	NA	(NA)	NA	(NA)	NA
Female	NA	(NA)	NA	(NA)	NA	NA	(NA)	NA	(NA)	NA
Age (years)										
65–74	1.000	(Ref.)	1.000	(Ref.)	Ref.	1.000	(Ref.)	1.000	(Ref.)	Ref.
40–64	0.442	(0.403 – 0.484)	0.591	(0.526 – 0.663)	<0.001	0.540	(0.495 – 0.590)	0.760	(0.676 – 0.855)	<0.001
20–39	0.597	(0.537 – 0.665)	0.566	(0.489 – 0.656)	<0.001	NA	(NA)	NA	(NA)	NA
Marital status										
Married	1.000	(Ref.)	1.000	(Ref.)	Ref.	1.000	(Ref.)	1.000	(Ref.)	Ref.
Single	2.269	(2.060 – 2.498)	2.929	(2.619 – 3.275)	<0.001	1.446	(1.264 – 1.654)	1.573	(1.363 – 1.815)	<0.001
Divorced/widowed	1.532	(1.370 – 1.714)	1.275	(1.131 – 1.437)	<0.001	1.446	(1.288 – 1.624)	1.238	(1.094 – 1.401)	0.001
Constant visit to hospitals †										
Yes (constant visit)	1.000	(Ref.)	1.000	(Ref.)	Ref.	1.000	(Ref.)	1.000	(Ref.)	Ref.
No (no-constant visit)	1.061	(0.988 – 1.140)	1.404	(1.292 – 1.526)	<0.001	1.084	(0.999 – 1.176)	1.470	(1.341 – 1.611)	<0.001
Subjective health status										
Good	1.000	(Ref.)	1.000	(Ref.)	Ref.	1.000	(Ref.)	1.000	(Ref.)	Ref.
Normal	1.251	(1.159 – 1.350)	1.163	(1.070 – 1.264)	<0.001	1.280	(1.173 – 1.397)	1.181	(1.075 – 1.296)	<0.001
Bad	1.192	(1.054 – 1.349)	1.048	(0.904 – 1.215)	0.532	1.420	(1.236 – 1.630)	1.194	(1.016 – 1.404)	0.032
Alcohol consumption										
Never or quit drinking	1.000	(Ref.)	1.000	(Ref.)	Ref.	1.000	(Ref.)	1.000	(Ref.)	Ref.
Social drinker/low-risk group (> 0 to ≤ 100 g/week)	0.743	(0.680 – .812)	0.827	(0.753 – 0.908)	<0.001	0.698	(0.631 – 0.772)	0.797	(0.718 – 0.886)	<0.001
Middle- risk drinking (> 100 to ≤ 350 g/week)	0.789	(0.693 – .897)	0.903	(0.788 – 1.036)	0.145	0.761	(0.661 – 0.876)	0.821	(0.707 – 0.953)	0.009
High-risk drinking (> 350 g/week)	1.027	(.744 – 1.417)	1.166	(0.832 – 1.633)	0.373	1.368	(0.951 – 1.968)	1.481	(1.014 – 2.162)	0.042
Smoking habit										
Never/ex-smoker	1.000	(Ref.)	1.000	(Ref.)	Ref.	1.000	(Ref.)	1.000	(Ref.)	Ref.
Current smoker	1.677	(1.454 – 1.934)	1.692	(1.454 – 1.969)	<0.001	2.024	(1.721 – 2.379)	2.004	(1.691 – 2.375)	<0.001
Educational qualification										

Vocational school/junior college/community(technical) college/university/post graduate school	1.000	(Ref.)	1.000	(Ref.)	Ref.	1.000	(Ref.)	1.000	(Ref.)	Ref.
High school	1.445	(1.342 – 1.557)	1.266	(1.168 – 1.374)	<0.001	1.527	(1.404 – 1.661)	1.284	(1.174 – 1.404)	<0.001
Primary/junior high school	2.591	(2.161 – 3.106)	1.669	(1.374 – 2.029)	<0.001	2.572	(2.132 – 3.104)	1.584	(1.295 – 1.937)	<0.001
Subjective financial state										
Wealthy	1.000	(Ref.)	1.000	(Ref.)	Ref.	1.000	(Ref.)	1.000	(Ref.)	Ref.
Nor poor not wealthy	1.178	(1.009 – 1.375)	1.058	(0.900 – 1.245)	0.493	1.225	(1.025 – 1.465)	1.129	(0.938 – 1.359)	0.201
Poor	1.539	(1.321 – 1.793)	1.345	(1.144 – 1.581)	<0.001	1.677	(1.406 – 2.001)	1.475	(1.225 – 1.774)	<0.001
Kessler Psychological Distress Scale										
Normal(total score =< 4)	1.000	(Ref.)	1.000	(Ref.)	Ref.	1.000	(Ref.)	1.000	(Ref.)	Ref.
Mild illness (5 ≦ total score ≦ 12)	0.947	(0.870 – 1.032)	0.904	(0.822 – 0.993)	0.036	0.939	(0.852 – 1.036)	0.888	(0.798 – 0.989)	0.031
Severe illness (13 ≦ total score)	1.076	(0.905 – 1.279)	0.870	(0.715 – 1.060)	0.168	1.485	(1.185 – 1.861)	1.294	(1.009 – 1.660)	0.042
Health insurance										
Employee insurance	1.000	(Ref.)	1.000	(Ref.)	Ref.	1.000	(Ref.)	1.000	(Ref.)	Ref.
National Health Insurance	2.122	(1.957 – 2.301)	1.468	(1.321 – 1.632)	<0.001	2.031	(1.862 – 2.214)	1.449	(1.294 – 1.623)	<0.001
Other	3.269	(2.352 – 4.543)	1.735	(1.226 – 2.456)	0.002	4.720	(3.280 – 6.792)	2.517	(1.719 – 3.687)	<0.001
Employment status										
Employed	1.000	(Ref.)	1.000	(Ref.)	Ref.	1.000	(Ref.)	1.000	(Ref.)	Ref.
Self-employed	1.272	(1.030 – 1.572)	0.958	(0.762 – 1.205)	0.716	1.620	(1.292 – 2.032)	1.232	(0.967 – 1.569)	0.091
Employed(other)	1.358	(1.173 – 1.571)	1.210	(1.032 – 1.419)	0.019	1.643	(1.403 – 1.925)	1.395	(1.175 – 1.656)	<0.001
Unemployed	1.866	(1.722 – 2.021)	1.482	(1.343 – 1.634)	<0.001	1.933	(1.769 – 2.112)	1.473	(1.320 – 1.644)	<0.001
Model chi square test			P<0.001					P<0.001		
Hosmer-Lemeshow test			P=0.035					P=0.007		

Note: OR, odds ratio; CI, confidence interval; Ref., reference; NA, not available

† No clear definition regarding the frequency of visit is provided in the questionnaire.
